# Pretreatment Radiologically Enlarged Lymph Nodes as a Significant Prognostic Factor in Clinical Stage IIB Cervical Cancer: Evidence from a Taiwanese Tertiary Care Center in Reaching Consensus

**DOI:** 10.3390/diagnostics12051230

**Published:** 2022-05-14

**Authors:** Chia-Hao Liu, Szu-Ting Yang, Wei-Ting Chao, Jeff Chien-Fu Lin, Na-Rong Lee, Wen-Hsun Chang, Yi-Jen Chen, Peng-Hui Wang

**Affiliations:** 1 Department of Obstetrics and Gynecology, Taipei Veterans General Hospital, Taipei 112, Taiwan; chliu12@vghtpe.gov.tw (C.-H.L.); styang6@vghtpe.gov.tw (S.-T.Y.); wtchao@vghtpe.gov.tw (W.-T.C.); nllee@vghtpe.gov.tw (N.-R.L.); whchang@vghtpe.gov.tw (W.-H.C.); chenyj@vghtpe.gov.tw (Y.-J.C.); 2 Department of Obstetrics and Gynecology, National Yang Ming Chiao Tung University, Taipei 112, Taiwan; 3 Institute of Clinical Medicine, National Yang Ming Chiao Tung University, Taipei 112, Taiwan; 4 Department of Statistics, National Taipei University, Taipei 112, Taiwan; cflin@gm.ntpu.edu.tw; 5 Department of Orthopedic Surgery, Wan Fang Hospital, Taipei Medical University, Taipei 116, Taiwan; 6 Department of Nursing, Taipei Veterans General Hospital, Taipei 112, Taiwan; 7 Department of Medical Research, China Medical University Hospital, Taichung 404, Taiwan; 8 Female Cancer Foundation, Taipei 112, Taiwan

**Keywords:** cervical cancer, clinical stage IIB, enlarged lymph nodes, radiologic imaging, progression-free survival, overall survival

## Abstract

The incidence of lymph node (LN) involvement and its prognostic value based on radiological imaging in stage IIB cervical cancer (CC) remains unclear, and evidence regarding oncological outcomes of patients with stage IIB CC with LN metastases is limited. In this study we retrospectively reviewed the incidence and prognostic significance of pretreatment radiologic LN status in 72 patients with clinical stage IIB CC (FIGO 2009), with or without radiologic evidence of LN enlargement. An enlarged LN was defined as a diameter > 10 mm on CT/MRI. Progression-free survival (PFS) and overall survival (OS) were assessed. Radiologic LN enlargement of >10 mm was observed in 45.8% of patients with stage IIB CC. PFS (*p* = 0.0088) and OS rates (*p* = 0.0032) were significantly poorer in the LN group (*n* = 33) than in the non-LN group (*n* = 39). Univariate Cox analysis revealed that LN > 10 mm contributed to a higher rate of recurrence and mortality. In conclusion, nearly half of the patients with clinical stage IIB CC had enlarged LNs (>10 mm) identified during pretreatment radiologic evaluation, which negatively impacted prognosis. Our findings highlight the need to incorporate CT- or MRI-based LN assessment before treatment for stage IIB CC.

## 1. Introduction

Cervical cancer (CC) is the fourth most common cancer in women worldwide, with an estimated global incidence of 570,000 and 311,000 corresponding tumor-related deaths reported in 2018 [1,2]. Despite recent advances in various treatments for locally advanced CC, recurrence and mortality rates remain high [3,4,5,6]. Stage IIB CC is defined as cervical carcinoma that invades the parametrium and does not extend to the lower third of the vagina or pelvic wall [7]. Before the 2018 FIGO classification update, CC was clinically staged based on pelvic examination, without the need for radiologic imaging evaluation [8,9]. Since 2018, FIGO has incorporated CT/MRI-based image analysis to assess the extent of the lymphatic spread, and patients with histologically proven CC with pelvic lymph node (LN) metastasis and para-aortic LN metastasis have been upstaged to FIGO stage IIIC1 and IIIC2, respectively [7,8,9].

Concurrent chemoradiotherapy (CCRT) is the main recommended treatment strategy for locally advanced disease [10]. However, CCRT remains a therapeutic challenge in countries where access to advanced radiological imaging and radiotherapy is limited. Nevertheless, stage IIB CC may be selectively treated with neoadjuvant chemotherapy followed by radical hysterectomy to reduce the need for postoperative radiotherapy [2,3,6,11,12].

To date, limited studies have evaluated oncological outcomes in patients with clinical stage IIB CC with LN enlargement, and no clear consensus has been reached regarding the prognostic value and anatomical level of LN involvement in stage IIB CC [13,14,15]. Thus, we aimed to validate the prognostic outcomes of patients with stage IIB disease (FIGO 2009), with or without pretreatment radiological LN enlargement.

## 2. Materials and Methods

### 2.1. Patients

A computerized search was conducted for women diagnosed with FIGO 2009 clinical stage IIB CC at Taipei Veterans General Hospital between January 2000 and December 2017 (Institutional Review Board protocol number: 2020-03-003AC). Medical records were retrospectively reviewed. In our gynecologic oncology clinic, CC is routinely staged by performing pelvic examination. Histopathological evaluation was performed according to the 2014 World Health Organization (WHO) criteria [16]. Cervical tumors were classified as squamous cell carcinoma, adenocarcinoma, or others (neuroendocrine or poorly differentiated carcinoma).

### 2.2. Radiologic Image Assessment

After the pathological confirmation, patients were radiologically assessed using an abdominal and pelvic MRI or a computed tomography (CT) before treatment initiation. Tumor size was defined as the largest tumor diameter on radiologic images. Size was the main criterion used to diagnose nodal involvement, with LN > 10 mm in the short axis [17,18,19]. The distribution and extent of LN involvement were categorized according to the corresponding anatomy based on the 2008 Querleu–Morrow classification (Level I, external iliac, internal iliac, and obturator regions; Level II, common iliac region; and Level III, para-aortic region) [20].

### 2.3. Treatment

The treatment strategy was selected after reaching a consensus at a multidisciplinary conference, according to the institutional gynecologic oncology guidelines. All patients who received chemotherapy underwent careful evaluation using the following criteria: WHO performance status of 0–2; adequate bone marrow reserve (absolute granulocyte count ≥ 2000/mL, platelet count ≥ 100,000/mL, and hemoglobin ≥ 10 g/dL); and adequate hepatic, pulmonary, and cardiac function. All radical hysterectomy (RH) procedures performed in this study were type C1 hysterectomy with bilateral pelvic LN dissection and para-aortic lymph node (PALN) dissection, as defined by the updated classification by Querleu and Morrow [21].

### 2.4. Follow-Up

All patients were followed up by quarterly surveillance for the first 2 years, semi-annually for the next 3 years, and annually after 5 years following primary treatment. During follow-up, the patients were questioned about possible symptoms which were confirmed by routine pelvic examination, vaginal or cervical smears, complete blood count, renal function tests, and transvaginal ultrasonography. Serum tumor markers and appropriate imaging studies (MRI, CT, or PET-CT) were arranged for cases of suspected recurrence.

### 2.5. Data Analysis

Continuous variables are presented as mean and standard deviation (SD) and were compared using Student’s *t*-test. The normality test for continuous variables was performed using the Shapiro-Wilk’s test (Table S1). Categorical variables are presented as numbers and percentages and were compared using Fisher’s exact test. Progression-free survival (PFS) was defined as the period from the date of treatment initiation to the date of cancer recurrence or last contact. Overall survival (OS) was defined as the period from treatment initiation to death, related to any cause or last contact. PFS and OS probabilities were estimated and compared using the Kaplan–Meier (KM) method and log-rank test. The univariate Cox proportional hazards model was used to quantify the effect of risk on survival for each variable. Data were analyzed using R 4.1.0: a language and environment for statistical computing (R Foundation for Statistical Computing, Vienna, Austria; http://www.R-project.org/, accessed on 1 May 2022).

## 3. Results

### 3.1. Patients

A total of 81 patients with clinical stage IIB (FIGO 2009) CC underwent treatment at our institution between 2010 and 2017. We excluded six patients who were partially treated at other hospitals, one patient who discontinued treatment due to acute hepatitis, and two patients with insufficient follow-up time. Ultimately, 72 patients were included in the final analysis. A total of 33 (45.8%) patients exhibited radiologic evidence of LN enlargement (>10 mm), and 39 (54.2%) exhibited LN < 10 mm or normal size in the initial imaging evaluation. There were no statistically significant differences in patient age, radiological study methods, ECOG performance status, comorbidities, tumor histology, tumor differentiation, primary treatment, and follow-up time between the two groups. The baseline patient characteristics are presented in Table 1.

### 3.2. Survival

**Figure 1:**
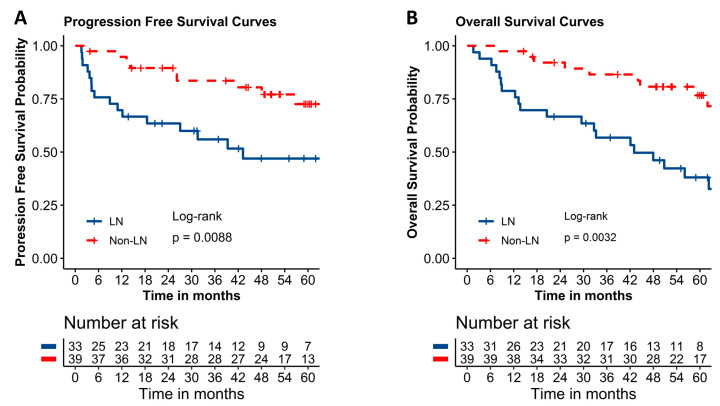
Kaplan–Meier curves depicting comparative 5-year PFS (**A**) and OS (**B**) between LN and non-LN groups of patients with stage IIB cervical cancer. LN: lymph node, OS, overall survival; PFS, progression-free survival.

The median follow-up time for the LN and non-LN groups was 2115 (48–3677) days and 1938 (254–3820) days, respectively (*p* = 0.829). The LN group (*n* = 33) had significantly poorer PFS and OS rates compared to the non-LN group (*n* = 39) (*p* = 0.0088 and *p* = 0.0032, respectively). Specifically, the 1-, 2-, and 5-year PFS rates (95% CI) were 69.7% (51.0–82.4), 63.5% (44.7–77.4), and 47.0% (27.9–64.0) for the LN group; 94.8% (80.8–98.7), 89.5% (74.5–95.9), and 72.6% (53.1–85.0) for the non-LN group, respectively (Figure 1A). OS also exhibited a similar trend. The 1-, 2-, and 5-year OS rates were 78.8% (60.6–89.3), 66.7% (47.9–80.0), and 38.1% (20.9–55.2) for the LN group; 97.4% (83.2–99.6), 92.1% (77.4–97.4), and 76.7% (58.3–87.8) for the non-LN group, respectively (Figure 1B). The PFS and OS rates are presented in Supplementary Tables S2 and S3, respectively.

### 3.3. Impact of LN Involvement on Recurrence and Survival

Univariate Cox analysis (Table 2) revealed that recurrence was directly influenced by LN metastatic level according to Querleu and Morrow. Level II (hazard ratio (HR), 3.48; 95% confidence interval (CI), 1.06−11.4; *p* = 0.039) and level III LN enlargement (HR, 29.7; 95% CI, 5.19 to 171; *p* < 0.001) contributed to significantly poorer PFS, leading to a higher risk of disease recurrence. Additionally, a radiologically enlarged LN identified at levels I and II contributed to a two- to three-fold higher risk of mortality (*p* = 0.031 and *p* = 0.013, respectively). Level III LN involvement was the most significant negative impact factor, increasing the risk of mortality by nearly seven-fold (HR, 6.71; 95% CI, 1.48−30.5; *p* = 0.014). Patients who received only definite RT had a higher risk of recurrence compared to other primary treatments in the LN group (HR, 3.36; 95% CI, 1.02−11.1; *p* = 0.046). No other notable risk factors were identified except LN enlargement, which negatively affected PFS and OS.

## 4. Discussion

In the present study, enlarged LN cases contributed to nearly three times higher risk of recurrence and four times higher mortality compared to cases without LN enlargement in stage IIB CC. Although previous studies have evaluated the prognostic implications of LN positivity in CC and demonstrated increased recurrence and decreased OS, a stage-specific analysis focusing solely on stage IIB disease is lacking [13,14,15]. The 5-year OS of the entire cohort of stage IIB CC was 57.4% in this study, which is lower than that of a population-based analysis, ranging from 63.5–65.8% in two study periods [22]. In the current Surveillance, Epidemiology, and End Results (SEER) database, the 5-year survival rate of all CC stages diagnosed between 2011 and 2018 was 66.7% [23]. In the subgroup analysis, the SEER database categorized patients with CC into localized, regional, and distant stages (Table 3), rather than based on the FIGO classification. The relative 5-year survival was 59.4% in the regional group, which was defined by the tumor spreading beyond the cervix to the regional LN [23], and is similar to our stage IIB data. These data imply that more advanced cases were included in the stage IIB CC cases at our institution due to inadequate staging via clinical staging.

Discrepancies exist between clinically and radiologically staged CC, which may significantly affect prognosis. Yoon et al. retrospectively compared the long-term outcomes of clinically and MRI-staged IIB CC and reported different 5-year OS rates between clinically and MRI-staged CC [24]. In this study, a higher frequency of regional LN metastasis (45.8%) in our stage IIB cohort during the initial image evaluation may have resulted in a lower 5-year OS despite adequate treatment. Recent literature has indicated that the rate of regional LN metastasis is approximately 16–36% for stage IIB CC [25]. Previous studies have also demonstrated a lower rate of LN metastasis in stage IIB CC compared to the present study, ranging from 25.8–30% [26,27,28,29,30]. The frequency of regional LN metastasis in stage IIB CC reported in previous studies is presented in Table 4.

We demonstrated that LNs involving a higher anatomic level were associated with poor prognosis, as demonstrated by an increased rate of recurrence and mortality. Huang et al. reported that patients with stage IB-IIB CC with level II (common iliac lymph node metastasis) had a poorer prognosis compared to those with involvement of other pelvic sites [28]. In the LN group, level III LN involvement was observed in two patients. Univariate Cox analysis revealed a drastic increase in recurrence risk (HR, 29.7; 95% CI, 5.19−171; *p* < 0.001). This finding is supported by the fact that the presence of PALN metastasis increased the recurrence risk by more than two-fold (OR, 2.129; 95% CI: 1.011−4.485; *p* = 0.047) in patients with locally advanced disease (FIGO 2009, IB1–IIIB) [27]. Moreover, Kilic et al. demonstrated that PALN was the only independent prognostic factor for recurrence in early-stage or locally advanced CC [31]. Furthermore, the presence of PALN metastasis was significantly associated with distant recurrence [31]. These studies confirmed the prognostic significance of level III LN status.

In this study, 72.7% of patients with clinical stage IIB CC in the LN group underwent an MRI as an initial evaluation, whereas 27.3% opted for a CT. However, the differences in radiological assessments did not affect the prognosis. The MRI is the method of choice for radiologic assessment of primary tumors with dimensions > 10 mm [32,33]. Notably, a meta-analysis by Scheidler et al. reported that a CT had a positive predictive value of 61% during pretreatment evaluation for the diagnosis of LN metastasis [34]. While the criterion for the detection of nodal metastasis is >10 mm, PET-CT has been reported to be more accurate than CT and MRI, resulting in only 4–15% of false-negative cases [35,36]. However, Hricak et al. compared pretreatment image evaluation methods for early invasive CC and observed similar staging accuracy between MRI and CT using surgical pathologic findings as the reference standard [32]. Collectively, these findings indicate that the MRI has a well-established role in defining the local extent of the primary tumor and is the modality of choice for preoperative staging and follow-up in patients with CC [17,37,38]. FDG PET/CT can improve staging accuracy by the depiction of LN metastases [19].

The number of positive LNs adversely influences CC prognosis. Wang et al. reported that in CCRT-treated patients with CC, the radiological number of positive pelvic LNs (≥3) was an independent prognostic factor [39]. However, in the current study, we did not assess the number of LNs, as only nine patients with stage IIB CC from the LN group underwent RH. Despite the limited number of patients who underwent surgery, all patients with radiologically enlarged LNs who eventually received RH with lymphadenectomy were pathologically positive in the dissected nodes.

In the current study, patients with stage IIB CC who underwent definite RT exhibited significantly higher rates of recurrence (HR, 3.36; 95% CI, 1.02−11.1; *p* = 0.046) compared with other treatments. Currently, CCRT is the main recommended treatment for locally advanced CC [10,27,39]. Various clinical trials have demonstrated that CCRT results in a decrease in mortality compared with RT alone [7,10]. A large population-based study in Canada confirmed that CCRT was superior to RT alone [40]. CCRT is well tolerated by most patients, except for minor gastrointestinal and hematologic side effects. However, it may lead to irreversible late side effects including potential injury to the mucosa of the bladder, rectum, bowel, and other adjacent organs [41]. The risk of major complications due to RT, such as fistula or fibrosis, is related to the volume, total dose, dose per fraction, and radiosensitivity of the involved tissue [10]. Despite possible adverse effects, we strongly agree with the current guideline that CCRT should be the prior treatment option for locally advanced CC, especially in patients with LN-positive IIB CC.

The new 2018 staging system now requires radiologic evaluation of LNs with added benefits [7,10,19]. It enables the identification of enlarged LNs and the upstaging of locally advanced CC, leading to improved survival and circumventing additional surgery. However, Wright et al. argued that the classification of all women with positive LNs into stage III may result in a highly heterogeneous group of patients with greatly divergent survival rates [14].

The strength of this study is its stage-specific analysis that primarily involved MRI-based pretreatment radiological evaluation. We demonstrated that the frequency of LN enlargement in CC, on CT/MRI recorded from a large tertiary center in Taiwan, was 45.8%. In the absence of larger studies focusing on stage IIB CC, these results are valuable for reaching a consensus to effectively understand the prognostic outcomes of clinical stage IIB CC patients with pretreatment LN involvement. This study also has several limitations, such as the small sample size and single-center setting. Furthermore, since not all patients underwent surgery, the pathological and actual number of nodal positivity was not assessed. However, all patients in the LN enlargement group who subsequently underwent RH with lymphadenectomy exhibited pathological evidence of LN metastasis. Despite the inherent limitations, the data presented in the current study is worthy of further investigation and a larger population-based study is necessary to confirm our findings.

## 5. Conclusions

This study confirmed that radiologically enlarged LNs were associated with significantly poorer survival outcomes compared to cases without clinical stage IIB CC. The decreased survival in LN-positive stage IIB CC further validates the 2018 FIGO staging system incorporating CT- and/or MRI-based LN assessments. Consequently, patients with clinical stage IIB CC with radiologically enlarged LNs > 10 mm should be upstaged and treated with CCRT.

## Figures and Tables

**Table 1:** Demographic and clinicopathological characteristics of patients with clinical stage IIB CC.

	LN *n* = 33	Non-LN *n* = 39	*p*
**Age at diagnosis (years) (mean [SD])**	61.33 (13.17)	63.15 (17.04)	0.619
**Comorbidity (%)**			
Yes	3 (9.1)	9 (23.1)	0.203
No	30 (90.9)	30 (76.9)	
**Method of radiologic i** **maging (%)**			
MRI	24 (72.7)	25 (64.1)	0.460
CT	9 (27.3)	14 (35.9)	
**ECOG performance status (%)**			
0	11 (33.3)	9 (23.1)	0.473
1	22 (66.7)	27 (69.2)	
2	0 (0.0)	2 (5.1)	
3	0 (0.0)	1 (2.6)	
**Anatomical level of LN involved (%)**			
None	0 (0.0)	39 (100.0)	<0.001
Level I	23 (69.7)	0 (0.0)	
Level II	8 (24.2)	0 (0.0)	
Level III	2 (6.1)	0 (0.0)	
**Tumor histology (%)**			
SCC	28 (84.8)	35 (89.7)	0.818
Adenocarcinoma	2 (6.1)	3 (7.7)	
Adenosquamous	1 (3.0)	0 (0.0)	
Neuroendocrine	1 (3.0)	0 (0.0)	
Poorly differentiated carcinoma	1 (3.0)	1 (2.6)	
**Tumor differentiation (%)**			
Moderate	29 (87.9)	34 (87.2)	1.000
Poor	4 (12.1)	5 (12.8)	
**Tumor size (mm) (mean [SD])**	49.37 (17.25)	42.22 (13.04)	0.075
**Primary treatment (%)**			
RH	9 (27.3)	9 (23.1)	0.604
NACT+RH	2 (6.1)	4 (10.3)	
CCRT+RH	3 (9.1)	3 (7.7)	
CCRT	14 (42.4)	21 (53.8)	
Definite RT	5 (15.2)	2 (5.1)	
**NACT (%)**			
Yes	2 (6.1)	4 (10.3)	0.681
No	31 (93.9)	35 (89.7)	
**RH (%)**			
Yes	14 (42.4)	16 (41.0)	1.000
No	19 (57.6)	23 (59.0)	
**CCRT (%)**			
Yes	17 (51.5)	25 (64.1)	0.341
No	16 (48.5)	14 (35.9)	
**Median follow-up days (range)**	2115 (48, 3677)	1938 (254, 3820)	0.829

LN, lymph node; ECOG, Eastern Cooperative Oncology Group; PFS, progression-free survival; OS, overall survival; SCC, squamous cell carcinoma; NACT, neoadjuvant chemotherapy; RH, radical hysterectomy; CCRT, concurrent chemoradiotherapy.

**Table 2:** Univariate Cox analysis of PFS and OS.

Covariate	PFS			OS		
	HR	95% CI	*p*	HR	95% CI	*p*
**LN enlarged > 10 mm**						
No	1.00			1.00		
Yes	2.86	1.26, 6.50	0.012	2.82	1.37, 5.78	0.005
**Age at diagnosis (years)**	1.01	0.98, 1.04	0.428	1.01	0.99, 1.03	0.477
**Comorbidity**						
No	1.00			1.00		
Yes	0.78	0.23, 2.61	0.686	2.45	1.09, 5.52	0.031
**Method of radiologic imaging**						
MRI	1.00			1.00		
CT	1.31	0.58, 2.97	0.517	1.23	0.57, 2.62	0.599
**ECOG performance status**			0.20			0.90
0	1.00			1.00		
1	0.59	0.25, 1.36	0.215	0.80	0.37, 1.70	0.562
2	2.65	0.57, 12.3	0.215	0.63	0.08, 4.94	0.660
3	0.00	0.00, Inf	0.997	0.00	0.00, Inf	0.997
**Anatomical level of LN involved**			0.006			0.020
None	1.00			1.00		
Level I	2.34	0.95, 5.77	0.065	2.38	1.08, 5.22	0.031
Level II	3.48	1.06, 11.4	0.039	3.81	1.32, 11.0	0.013
Level III	29.7	5.19, 171	<0.001	6.71	1.48, 30.5	0.014
**Tumor histology**			0.500			0.500
SCC	1.00			1.00		
Adenocarcinoma	1.71	0.40, 7.33	0.470	2.53	0.88, 7.30	0.086
Adenosquamous	2.93	0.39, 22.0	0.296	0.00	0.00, Inf	0.997
Neuroendocrine	2.55	0.34, 19.1	0.361	1.78	0.24, 13.3	0.572
Poorly differentiated carcinoma	0.00	0.00, Inf	0.998	1.06	0.14, 7.90	0.953
**Tumor differentiation**			0.800			0.980
Moderate	1.00			1.00		
Poor	0.84	0.25, 2.80	0.773	1.00	0.35, 2.87	0.994
**Tumor size (mm)**	1.02	0.99, 1.05	0.263	1.03	1.00, 1.05	0.056
**Primary treatment**			0.200			0.300
RH	1.00			1.00		
NACT+RH	0.45	0.05, 3.75	0.462	0.83	0.17, 4.00	0.816
CCRT+RH	0.95	0.19, 4.71	0.949	0.69	0.14, 3.36	0.650
CCRT	0.98	0.36, 2.65	0.969	1.06	0.43, 2.64	0.893
Definite RT	3.36	1.02, 11.1	0.046	2.57	0.90, 7.35	0.078
**NACT**						
No	1.00			1.00		
Yes	0.39	0.05, 2.90	0.359	0.71	0.17, 2.98	0.642
**RH**						
No	1.00			1.00		
Yes	0.69	0.31, 1.57	0.380	0.68	0.33, 1.40	0.295
**CCRT**						
No	1.00			1.00		
Yes	0.89	0.41, 1.97	0.780	0.84	0.42, 1.68	0.615

PFS, progression-free survival; OS, overall survival; LN, lymph node; ECOG, Eastern Cooperative Oncology Group; SCC, squamous cell carcinoma; NACT, neoadjuvant chemotherapy; CCRT, concurrent chemoradiotherapy.

**Table 3:** Five-year relative survival for different CC stages according to SEER database (2012–2018).

SEER Stage	5-Year Relative Survival (%)
Localized	91.8
Regional	59.4
Distant	17.1
Unknown	53.6

SEER: Surveillance, Epidemiology, and End Results.

**Table 4:** List of studies reporting frequency of LN metastasis associated with FIGO stage IIB.

Study	Frequency of Regional LN Metastasis in Stage IIB CC (%)
Handa et al. [25]	16–36
Sakuragi et al. [30]	25.5
Liu et al. [29]	25.8
Chen et al. [26]	27
Endo et al. [27]	29
Huang et al. [28]	30
The current study	45.8

LN: lymph node; CC: cervical cancer.

## Data Availability

The data supporting the findings of this study are available from the corresponding author upon reasonable request.

## Supplementary Materials

The following supporting information can be downloaded at: https://www.mdpi.com/article/10.3390/diagnostics12051230/s1, Table S1: Shapiro-Wilk’s test shows the normal distribution for all continuous variables; Table S2: The PFS rates of LN and non-LN groups from clinical stage IIB CC; Table S3: The OS rates of LN and non-LN groups from clinical stage IIB CC.
