# Supplementary material for: Pretreatment Radiologically Enlarged Lymph Nodes as a Significant Prognostic Factor in Clinical Stage IIB Cervical Cancer: Evidence from a Taiwanese Tertiary Care Center in Reaching Consensus
Source: Diagnostics (Basel). 2022 May 14;12(5):1230. doi: 10.3390/diagnostics12051230 (PMC9140083; doi:10.3390/diagnostics12051230)
Supplement: Supplementary file 1 [file diagnostics-12-01230-s001.zip › Table S1.pdf]

Table S1. Shapiro-Wilk's test shows the normal distribution for all continuous variables.

	Age w-value	Size w-value	Age p-value	Size p-value
Total	0.985	0.981	0.5524	0.501
LN	0.985	0.969	0.9176	0.585
non-LN	0.948	0.972	0.0682	0.542
