# Supplementary material for: Pretreatment Radiologically Enlarged Lymph Nodes as a Significant Prognostic Factor in Clinical Stage IIB Cervical Cancer: Evidence from a Taiwanese Tertiary Care Center in Reaching Consensus
Source: Diagnostics (Basel). 2022 May 14;12(5):1230. doi: 10.3390/diagnostics12051230 (PMC9140083; doi:10.3390/diagnostics12051230)
Supplement: Supplementary file 1 [file diagnostics-12-01230-s001.zip › Table S2.pdf]

Table S2. The PFS rates of LN and non-LN groups from clinical stage IIB CC.

PFS_time_mo	PFS_LN_Yes	PFS_LowCI95_LN_Yes	PFS_UpperCI95_LN_Yes	PFS_LN_No	PFS_LowCI95_LN_No	PFS_UpperCI95_LN_No
6	0.758	0.573	0.871	0.974	0.832	0.996
12	0.697	0.51	0.824	0.948	0.808	0.987
18	0.667	0.479	0.8	0.895	0.745	0.959
24	0.635	0.447	0.774	0.895	0.745	0.959
30	0.6	0.411	0.745	0.836	0.669	0.923
36	0.56	0.369	0.713	0.836	0.669	0.923
42	0.517	0.325	0.678	0.836	0.669	0.923
48	0.47	0.279	0.64	0.805	0.632	0.902
54	0.47	0.279	0.64	0.771	0.592	0.879
60	0.47	0.279	0.64	0.726	0.531	0.85
