# Supplementary material for: Pretreatment Radiologically Enlarged Lymph Nodes as a Significant Prognostic Factor in Clinical Stage IIB Cervical Cancer: Evidence from a Taiwanese Tertiary Care Center in Reaching Consensus
Source: Diagnostics (Basel). 2022 May 14;12(5):1230. doi: 10.3390/diagnostics12051230 (PMC9140083; doi:10.3390/diagnostics12051230)
Supplement: Supplementary file 1 [file diagnostics-12-01230-s001.zip › Table S3.pdf]

Table S3. The OS rates of LN and non-LN groups from clinical stage IIB CC.

OS_time_mo	OS_LN_Yes	OS_LowCI95_LN_Yes	OS_UpperCI95_LN_Yes	OS_LN_No	OS_LowCI95_LN_No	OS_UpperCI95_LN_No
6	0.939	0.779	0.984	1	1	1
12	0.788	0.606	0.893	0.974	0.832	0.996
18	0.697	0.51	0.824	0.921	0.774	0.974
24	0.667	0.479	0.8	0.921	0.774	0.974
30	0.635	0.447	0.774	0.893	0.739	0.959
36	0.568	0.381	0.718	0.865	0.705	0.942
42	0.568	0.381	0.718	0.865	0.705	0.942
48	0.462	0.281	0.624	0.807	0.637	0.903
54	0.423	0.247	0.59	0.807	0.637	0.903
60	0.381	0.209	0.552	0.767	0.583	0.878
